# Reliability of long vs short COI markers in identification of forensically important flies

**DOI:** 10.3325/cmj.2014.55.19

**Published:** 2014-02

**Authors:** Sanaa M. Aly

**Affiliations:** Department of Forensic Medicine and Clinical Toxicology, Faculty of Medicine, Suez Canal University, Ismailia, Egypt

## Abstract

**Aim:**

To compare the reliability of short and long cytochrome oxidase I gene fragment (COI) in identification of forensically important Diptera from Egypt and China.

**Methods:**

We analyzed 50 specimens belonging to 18 species. The two investigated markers were amplified by polymerase chain reaction (PCR) followed by direct sequencing. Nucleotide sequence divergences were calculated using the Kimura two-parameter (K2P) distance model and neighbor-joining (NJ) phylogenetic trees.

**Results:**

Although both tested fragments showed an overlap between intra and interspecific variations, long marker had greater completeness of monophyletic separation with high bootstrap support. Moreover, NJ tree based on the long fragment clustered species more in accordance with their taxonomic classification than that based on the short fragment.

**Conclusion:**

In dipterous identification, it is recommended to use the long COI marker due to its greater reliability and safety.

Necrophagous insects can serve as a valuable source of information for estimation of minimum post-mortem interval (PMI) in legal medicine. Most suitable for forensic purposes are species from the order Diptera (eg, Calliphoridae, Muscidae, and Sarcophagidae) (1-4). In PMI estimation, an important initial step is correct identification of these insects, which may be difficult by using the traditional morphology-based approach (5,6), because several forensically important fly species can hardly be distinguished morphologically (7-9). The limitations of morphological method can be overcome by gene sequences analysis, a fast and accurate method of species identification. Molecular analysis requires small tissue samples and is relatively insensitive to preservation conditions (1,10). Different mitochondrial (mt) and nuclear (nu) DNA markers are investigated as forensic tools. However, mtDNA is preferred because it can be easily extracted even from small or degraded samples (10). In addition, because of its strictly maternal inheritance and lack of genetic recombination, mtDNA haplotype is a good candidate for evolutionary and population genetics study.

Mitochondrial cytochrome c oxidase subunit I (COI) sequences are a rapid and powerful tool for accurate identification of species across various taxa (7,11-14). Although COI has been extensively studied by forensic entomologists, resulting in a vast amount of DNA data, there is little agreement as to which portion of the gene needs to be sequenced. Although the 5′ end of COI is also the site of the proposed universal animal DNA “barcode” (11) and it has been successfully used in the identification of many blowfly species (12), this approach cannot identify some closely related species (12,15). Therefore, to optimize discrimination power between closely related species some authors suggested multi-gene approach (16,17). Surprisingly, a recent study using this approach revealed that phylogenetic tree based on COI fragment was similar to that based on 3 different gene fragments (16).

Fragments of the COI sequence that show low sequence divergence within species but high divergences among species can be employed as taxon “barcodes,” and unknown samples can be accurately grouped to species with reference sequences of the “barcode library” (14,18,19). Therefore, it is paramount to evaluate not only discrimination power of these COI fragments between closely related species but also between species belonging to more than one family, because in a database an unknown sample will be compared to all reference samples. In the absence of an appropriate reference sample, unknown samples will simply group with the most closely matched reference sample (20). Thus, it is important to confirm that the investigated marker will not only be correctly assigned to a species but also that it will be in accordance with the traditional morphological classification. Therefore, we evaluated the discrimination power of the short (272-bp) COI fragment in the identification of the most forensically relevant flies (Calliphoridae, Sarcophagidae, and Muscidae) originating from Egypt and China in comparison to the long (1173-bp) COI fragment, and aimed to gather genetic data on common forensically important Diptera.

## Materials and methods

### Samples

Fifty adult flies belonging to 18 species including 10 species of Calliphoridae, 5 species of Sarcophagidae, and 3 species of Muscidae were collected during two consecutive years (1/2011 to 12/2012). This study was conducted in both Forensic Medicine & Clinical Toxicology department, Faculty of Medicine, Suez Canal University, Ismailia, Egypt and National Key Laboratory, Basic Medical School, Central south University, Changsha, China. All samples were collected using traps baited with animal remains. Collected flies were trapped at different locations in Egypt and China (Table 1). Samples were identified by entomologists based on traditional morphological characteristics (21-25). All samples were subsequently stored in 70% ethanol at -20°C. For comparison, other sequences were retrieved from the NCBI database (*http://www.ncbi.nlm.nih.gov*).

**Table 1:** Specimens used in the study

Species	Code in neighbor-joining tree	Location	Accession number			
***Chrysomya megacephala*** (Fabricius,1794)	CmC1	Changsha, China	KC249623			KC249673
	CmC2	Changsha, China	KC249624			KC249674
	CmE3	Ismailia, Egypt	KC249625			KC249675
	CmE4	Ismailia, Egypt	KC249626			KC249676
	Cm5		JX187372*			
***C. albiceps*** (Wiedemann, 1819)	CalbE1	Alkantra shark, Egypt	KC249627			KC249677
	CalbE2	Alkantra shark, Egypt	KC249628			KC249678
	CalbE3	Alkantra shark, Egypt	KC249629			KC249679
	CalbE4	Ismailia, Egypt	KC249630			KC249680
	CalbE5	Ismailia, Egypt	KC249631			KC249681
	Calb6		AF083657*			
***C. rufifacies*** (Macquart, 1842)	CrC1	Changsha, China	KC249632			KC249682
	CrC2	Changsha, China	KC249633			KC249683
	Cr3		JX187383*			
***C. nigripes*** (Aubertin, 1932)	CnC1	Changsha, China	KC249634			KC249684
	CnC2	Changsha, China	KC249635			KC249685
	CnC3	Guangzhou, China	KC249636			KC249686
	CnC4	Guangzhou, China	KC249637			KC249687
***Aldrichina graham*** (Aldrich, 1930)	AgC1	Changsha, China	KC249638			KC249688
	AgC2	Guangzhou, China	KC249639			KC249689
***Lucilia sericata*** (Meigen, 1826)	LsC1	Changsha, China	KC249640			KC249690
	LsC2	Changsha, China	KC249641			KC249691
***L. bazini*** (Seguy, 1934)	LbC1	Zhangjiajie China	KC249642			KC249692
	LbC2	Zhangjiajie China	KC249643			KC249693
***L. caesar**** (Linnaeus, 1758)*	LcaC1	China	KC249644			KC249694
	LcaC2	China	KC249645			KC249695
***L. cuprina*** (Wiedemann, 1830)	LcuC1	Changsha China	KC249646			KC249696
	LcuC2	Changsha China	KC249647			KC249697
***L. porphyrina*** (Walker, 1856)	LpC1	Changsha China	KC249648			KC249698
	LpC2	Changsha China	KC249649			KC249699
***Musca domestica*** (Linnaeus, 1758)	MdE1	Alkantra shark, Egypt	KC249650			KC249700
	MdE2	Ismailia Egypt	KC249651			KC249701
	MdE3	Ismailia Egypt	KC249652			KC249702
***M. autumnalis*** (De Geer, 1776)	MaC1	Changsha, China	KC249653			KC249703
	MaC2	Changsha, China	KC249654			KC249704
	MaE3	Ismailia, Egypt	KC249655			KC249705
	MaE4	Alkantra shark, Egypt	KC249656			KC249706
	MaE5	Portsaid, Egypt	KC249657			KC249707
***Fannia canicularis*** (Linnaeus, 1761)	FcE1	Ismailia, Egypt	KC249658			KC249708
	FcE2	Ismailia, Egypt	KC249659			KC249709
	FcE3	Alkantra shark, Egypt	KC249660			KC249710
***Sarcophaga albiceps*** (Meigen, 1826)	SalbC1	Changsha China	KC249661			KC249711
	SalbC2	Changsha China	KC249662			KC249712
***S. dux*** (Thompson, 1869)	SdC1	Changsha China	KC249663			KC249713
	SdC2	Changsha China	KC249664			KC249714
***S. Africa*** (Wiedemann, 1824)	SaC1	Changsha, China	KC249665			KC249715
	SaC2	Xining, China	KC249666			KC249716
	SaC3	Changsha, China	KC249667			KC249717
	Sa4		JQ582120*			
***S. argyrostoma*** (Robineau-Desvoidy,1830)	SargyE1	Alkantra shark, Egypt	KC249668			KC249718
	SargyE2	Ismailia Egypt	KC249669			KC249719
	SargyE3	Ismailia Egypt	KC249670	KC249720		
	Sargy4		JQ582123*			
***S. peregrine*** (Robineau-Desvoidy,1830)	SperC1	Changsha, China	KC249671			KC249721
	SperC2	Changsha, China	KC249672		KC249722	

*GenBank accession numbers are given for previously published sequences.

### DNA extraction

MtDNA was extracted from all samples using Mini Tissue Kit (Qiagen, Hilden, Germany) according to the manufacturer’s protocol. To avoid possible contamination of fly DNA with DNA from ingested proteins and eggs of gut parasites, the thoracic muscle of each insect was used as the source of DNA, whereas the head and abdomen were retained for further analysis.

### PCR amplification and DNA sequencing

The 272-bp COI gene fragment was amplified using the primers 5′-CAGATCGAAATTTAAATACTTC-3′ and 5′-GTATCAACATCTATTCCTAC-3′ and 1173-bp COI fragment was amplified using 5′ TACAATTTATCGCCTAAACTTCAGCC 3′ and 5′ CAGCTACTTTATGAGCTTTAGG 3′. Details of the primers and PCR condition were described in previous studies (26,27). Gel electrophoresis was used to isolate PCR products, which were then purified using QiaQuick PCR Purification Kit (Qiagen, Germantown, MD, USA). Column cycle sequencing was performed on both forward and reverse strands using ABI PRISM Big Dye Terminator Cycle Sequencing Ready Reaction Kit by ABI PRISM 3730 (Applied Biosystems, Foster City, CA, USA) with Big Dye terminator v. 3.1 as the sequencing agent.

### Sequences analysis and phylogenetic tree construction

Analysis of DNA sequence variations, nucleotide composition, and genetic distances analysis was performed using Molecular Evolutionary Genetics Analysis v. 5.10 (MEGA) (28). Phylogenetic trees based on the 2 investigated COI sequences were constructed by neighbor-joining (NJ) method using Kimura two-parameter (K2P) model implemented in the MEGA and tested by 1000 bootstrap replicates.

## Results

Both 272-bp and 1173-bp COI fragments were successfully sequenced from all 50 insects. The 272-bp and 1173-bp sequences corresponded to positions 2098-2369 and 1513-2685, respectively of Drosophila yakuba (GenBank accession number X03240).

**Figure 1:**
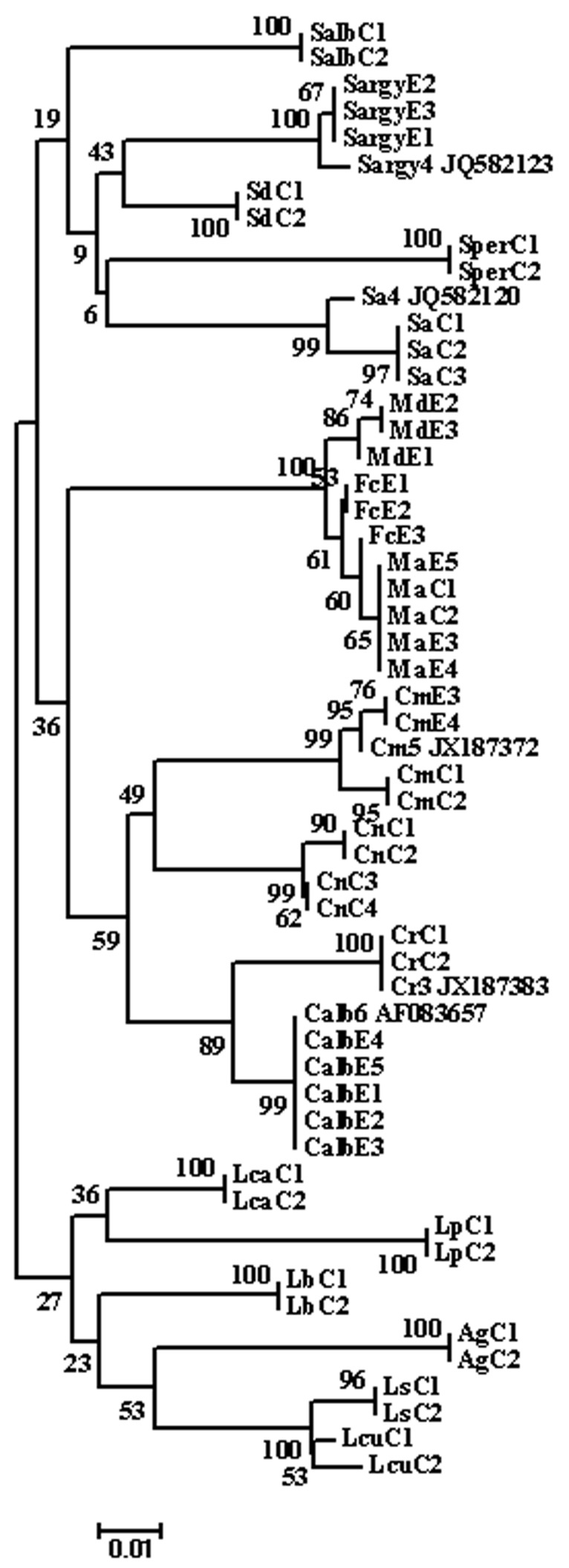
The neighbor-joining tree using Kimura’s 2-parameter model illustrating phylogenetic relationships among 18 fly species based on 272-bp cytochrome oxidase I sequences. Sample codes are as in Table 1. Numbers on branches indicate the support value. Evolutionary distance divergence scale bar is 0.01.

Based on 272-bp sequences, 73 were variant and 71 were parsimony-informative characters. The nucleotide composition showed much higher frequencies of adenine and thymine (31.7% and 37% of total nucleotide compositions, respectively) compared with 14.2% of cytosine and 17.1% of guanine. NJ analysis was conducted to determine the relationships between the analyzed species (Figure 1 and Table 2). All species were monophyletic with bootstrap support of 99%-100%, except *M. autumnalis* and *F. canicularis.* Both species could not be separated forming one polytypic clade with 61% support. Although Muscidae formed a distinct group with high bootstrap support (100%), 272-bp COI marker failed to distinguish between Muscidae and Fanniidae. Sarcophagidae family formed a distinct group but with low bootstrap support (19%). Calliphoridae family failed to form a distinct group. At the genus level, Lucilia formed a distinct group with 27% support. Although *Aldrichina grahami* belongs to Aldrichina genus, it was embedded within Lucilia group. Chrysomya group did not join directly with the other group (Lucilia) that belongs to the same family. All tested species displayed intraspecific variations from 0 to 1.5% (Table 2). The highest variation was observed in *C. megacephala* and *S. africa* at 1.5%. Although *M. autumnalis* samples were collected from 2 countries, 0% intraspecific variation was observed. The interspecific variations between 18 tested species varied from 1% to 14%. The minimum interspecific variations were between *M. Domestica, M. autumnalis, and F. canicularis* at 1%.

**Table 2:** Calculated intra- and interspecific divergences expressed as percentage of the analyzed 272-bp (below the diagonal) and 1173-bp (above the diagonal) cytochrome oxidase I gene fragment using neighbor-joining (NJ) approach with Kimura’s 2-parameter (K2P) model*

No	Species	N	V1	V2	1	2	3	4	5	6	7	8	9	10	11	12	13	14	15	16	17	18
**1**	***C. megacephala***	**4**	**0-1.5**	**0-1.5**	**-**	**6**	**8**	**6**	**11**	**10**	**9**	**9**	**9**	**10**	**10**	**11**	**11**	**12**	**12**	**13**	**13**	**13**
**2**	***C. albiceps***	**5**	**0**	**0-0.7**	**7**	**-**	**4**	**7**	**11**	**10**	**9**	**10**	**11**	**10**	**12**	**12**	**12**	**12**	**11**	**13**	**12**	**13**
**3**	***C. rufifacies***	**2**	**0**	**0-1.2**	**10**	**3**	**-**	**9**	**12**	**11**	**11**	**12**	**12**	**12**	**12**	**12**	**12**	**12**	**11**	**13**	**12**	**14**
**4**	***C. nigripes***	**4**	**0**	**0-0.5**	**7**	**5**	**7**	**-**	**10**	**10**	**10**	**10**	**10**	**11**	**11**	**11**	**11**	**12**	**11**	**14**	**13**	**14**
**5**	***A.grahami***	**2**	**0**	**0**	**12**	**11**	**11**	**11**	**-**	**9**	**8**	**8**	**8**	**9**	**14**	**14**	**14**	**12**	**12**	**14**	**13**	**13**
**6**	***L. bazini***	**2**	**0**	**0**	**10**	**8**	**9**	**8**	**8**	**-**	**6**	**7**	**7**	**7**	**12**	**12**	**12**	**11**	**11**	**12**	**12**	**12**
**7**	***L. caesar***	**2**	**0**	**0**	**8**	**8**	**8**	**10**	**9**	**7**	**-**	**6**	**6**	**5**	**11**	**11**	**10**	**11**	**12**	**11**	**12**	**12**
**8**	***L. cuprina***	**2**	**1**	**1**	**9**	**10**	**12**	**9**	**8**	**8**	**7**	**-**	**1**	**6**	**12**	**12**	**12**	**11**	**10**	**11**	**11**	**12**
**9**	***L. sericata***	**2**	**0**	**0**	**10**	**10**	**12**	**10**	**9**	**7**	**8**	**2**	**-**	**7**	**12**	**12**	**12**	**11**	**11**	**12**	**12**	**12**
**10**	***L. porphyrina***	**2**	**0**	**0**	**11**	**10**	**12**	**14**	**11**	**9**	**7**	**10**	**10**	**-**	**12**	**12**	**12**	**12**	**12**	**12**	**13**	**13**
**11**	***M. autumnalis***	**5**	**0**	**0-0.1**	**9**	**9**	**10**	**10**	**14**	**10**	**9**	**11**	**11**	**12**	**-**	**1**	**1**	**13**	**14**	**14**	**14**	**14**
**12**	***M. domestica***	**3**	**0-0.4**	**0-0.8**	**10**	**9**	**10**	**9**	**13**	**10**	**9**	**12**	**12**	**13**	**2**	**-**	**1**	**14**	**14**	**14**	**14**	**15**
**13**	***F. canicularis***	**3**	**0-0.4**	**0-0.6**	**10**	**8**	**9**	**9**	**13**	**10**	**9**	**11**	**12**	**13**	**1**	**1**	**-**	**13**	**13**	**13**	**14**	**14**
**14**	***S. albiceps***	**2**	**0**	**0**	**11**	**9**	**9**	**11**	**12**	**9**	**9**	**9**	**9**	**12**	**9**	**10**	**9**	**-**	**7**	**10**	**10**	**8**
**15**	***S. dux***	**2**	**0**	**0**	**11**	**6**	**9**	**8**	**11**	**7**	**9**	**8**	**9**	**12**	**9**	**9**	**8**	**7**	**-**	**9**	**8**	**8**
**16**	***S. argyrostoma***	**3**	**0-0.7**	**0-1**	**12**	**12**	**12**	**12**	**11**	**9**	**9**	**11**	**12**	**12**	**10**	**10**	**9**	**9**	**6**	**-**	**10**	**9**
**17**	***S. africa***	**3**	**0-1.5**	**0-2**	**12**	**10**	**9**	**9**	**10**	**10**	**9**	**11**	**12**	**12**	**12**	**12**	**12**	**9**	**7**	**9**	**-**	**9**
**18**	*S. peregrina*	**2**	**0**	**0**	**13**	**11**	**14**	**14**	**12**	**9**	**11**	**10**	**10**	**10**	**12**	**13**	**12**	**10**	**8**	**9**	**11**	**-**

*Abbreviations: N – number of specimens; V1 – intraspecific variations within 272-bp fragment; V2 – intraspecific variations within 1173-bp fragment.

**Figure 2:**
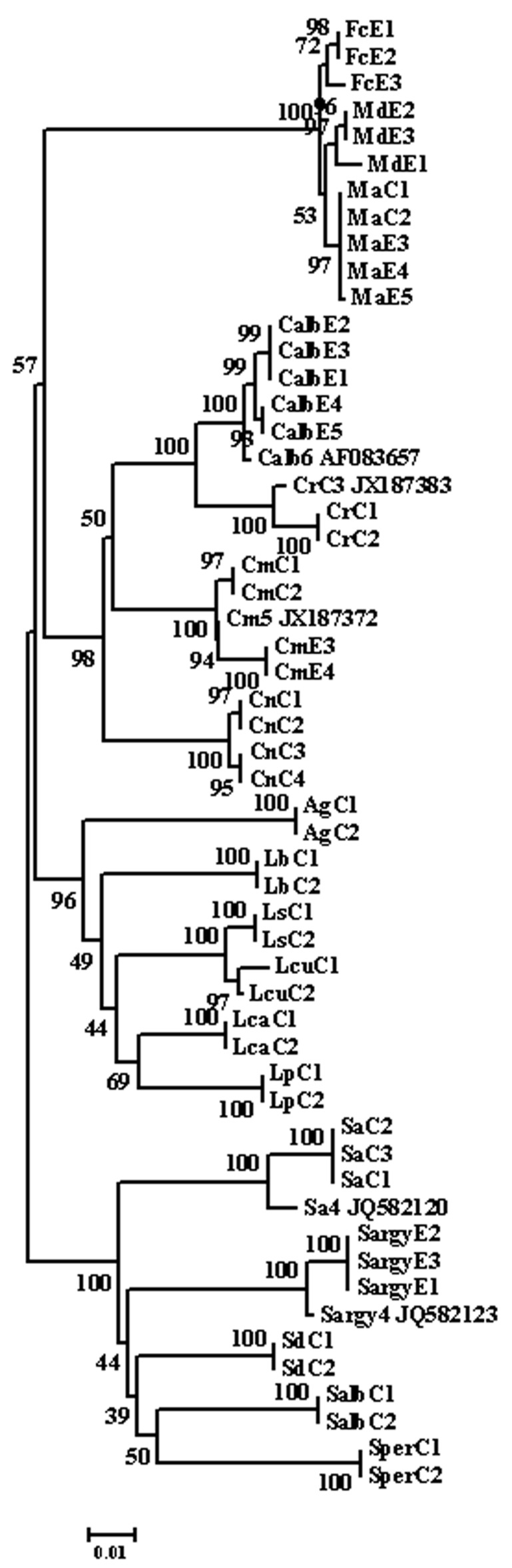
The neighbor-joining tree using Kimura’s 2-parameter model illustrating phylogenetic relationships among 18 fly species based on 1173-bp cytochrome oxidase I sequences. Sample codes are as in Table 1. Numbers on branches indicate the support value. Evolutionary distance divergence scale bar is 0.01.

Based on 1173-bp sequences, 386 were variant and 372 were parsimony-informative characters. The nucleotide composition showed much higher frequencies of adenine and thymine (29.9% and 38.8%, of total nucleotide composition, respectively), compared with 15.3% of cytosine and 16.1% of guanine. All tested species were monophyletic with full bootstrap supports (Figure 2 and Table 2). Sarcophagidae formed a distinct group with 100% bootstrap support. In the Muscidae group, 2 tested families (Muscidae/Fanniidae) could be separated. Calliphoridae family failed to form a distinct group. At the genus level, Lucilia formed a distinct group with 49% support. Aldrichina grahami, belonging to the Aldrichina genus, first formed a separate group then joined with that of Lucilia with 96% support. Chrysomya formed a group with 98% support. Interestingly, Chrysomya group joined with Muscidae before joining with other Calliphoridae (Lucilia and Aldrichina). All tested species displayed intraspecific variations ranging from 0 to 2% (Table 2). The highest level was observed for *S. africa* at 2%. Although samples were collected from 2 countries, 0% intraspecific variations were observed for *M. autumnalis.* The interspecific variations between 18 tested species varied from 1% to 15%. The minimum interspecific variations were found between *L. cuprina/L. sericata* and *M. domestica/M. autumnalis/F. canicularis* at 1%.

## Discussion

This study found that although both tested fragments showed an overlap between intra and interspecific variations, long marker showed greater completeness of monophyletic separation with high bootstrap support. To our knowledge, this is the first study to provide molecular data on forensically important species from Egypt and China by using either short 272-bp or long 1173-bp fragment of the mt COI gene. The mt COI gene has been shown to be a major candidate gene for identification of forensically important insects (7,14,27,29). So, before using it in real forensic entomology cases, it is worth evaluating the applicability of different 272-bp and 1173-bp COI genetic markers by using species from the specific geographic areas (30).

As expected, this region of mtDNA had a strong adenine-thymine bias, which is characteristic of insect mtDNA (6,12). No insertions or deletions were identified within the aligned sequences, as was found in studies conducted on other mtDNA fragments (6,11,31,32). Based on both tested COI fragments, *C. megacephala* and *M. autumnalis* samples were both sequenced from China and Egypt and showed minimal variation between populations. However, the largest intraspecific variation was observed between the species collected from different locations within one country. These results are in agreement with the study by Harvey et al (20), who tested 1167-bp COI for identification of Calliphoridae of Australian and South African origin. The low intraspecific variation between two countries indicates the value of the mtDNA region in interspecific distinction (33,34).

One study suggested that intraspecific variation should be ≤1% and between-species separation ≥3% (35), whereas other studies suggested establishing group-specific thresholds (8,11). In the present study, results of both short and long COI fragments support the idea of establishing group-specific thresholds because the 3 investigated species that belong to Muscidae exhibited the lowest interspecific variation, leading to an overlap between intraspecific and interspecific nucleotide divergences. Interestingly, although low sequence divergence can result in similar haplotypes, which may lead to misidentification and a wrong PMI estimate (8), 1173-bp COI was able to distinguish between *M. autumnalis* and *F. canicularis* without bias, but 272-bp COI was not.

Based on 1173-bp COI gene tree, all species were reciprocally monophyletic with full bootstrap support. This observation was the same as for the analysis based on 272-bp COI fragment, except for *M. autumnalis* and *F. canicularis*. Surprisingly, trees based on both fragments showed that Chrysomya clade did not directly join with the other clade belonging to Calliphoridae. This observation may shed light on the importance of examining the exact relationship between these groups.

Based on 1173-bp COI gene tree, Aldrichina clade presented a deviation from traditional taxonomy because this species (Calliphorinae) was identified as a sister species to Chrysomya rather than to Lucilia (16). This pattern of evolution was also observed previously based on 28rRNA alone (36) and based on COI, CYTB, and ITS2 in a multi-gene approach (16). This relation was different from that observed based on 272-bp COI, when *A. grahami* was embedded within Lucilia tribe. The data obtained by 1173-bp COI phylogenetic analysis were more in accordance with the traditional morphological classification than the data obtained by 272-bp COI fragment analysis.

In this preliminary genetic identification of fly species from Egypt and China, we found that the long COI fragment outperformed the short one in species identification. Since the sample size was small, we recommend an evaluation of more samples using the same and other loci to confirm our findings. In addition, it is important to identify additional forensically important fly species and expand such analyses to all relevant Egyptian and Chinese species.
